# B7-H3 Expression in Breast Cancer and Brain Metastasis

**DOI:** 10.3390/ijms25073976

**Published:** 2024-04-03

**Authors:** Vaibhavi Joshi, Kate Beecher, Malcolm Lim, Andrew Stacey, Yufan Feng, Parmjit S. Jat, Pascal H. G. Duijf, Peter T. Simpson, Sunil R. Lakhani, Amy E. McCart Reed

**Affiliations:** 1 UQ Centre for Clinical Research, Faculty of Medicine, The University of Queensland, Brisbane 4029, Australia; vaibhavi.joshi@uq.edu.au (V.J.); kate.matthews@uq.edu.au (K.B.); malcolm.lim@uq.edu.au (M.L.); andrew.stacey@health.qld.gov.au (A.S.); yufan.feng@uq.edu.au (Y.F.); p.simpson@uq.edu.au (P.T.S.); 2 MRC Prion Unit at UCL, Institute of Prion Diseases, Courtauld Building, London W1W 7FF, UK; p.jat@prion.ucl.ac.uk; 3 Centre for Cancer Biology, Clinical and Health Sciences, University of South Australia & SA Pathology, Adelaide 5001, Australia; pascal.duijf@unisa.edu.au; 4 Pathology Queensland, Royal Brisbane and Women’s Hospital, Brisbane 4029, Australia

**Keywords:** breast cancer, brain metastasis, biomarker, B7-H3, CD276, prognosis, therapeutic target

## Abstract

Brain metastasis is a significant challenge for some breast cancer patients, marked by its aggressive nature, limited treatment options, and poor clinical outcomes. Immunotherapies have emerged as a promising avenue for brain metastasis treatment. B7-H3 (CD276) is an immune checkpoint molecule involved in T cell suppression, which is associated with poor survival in cancer patients. Given the increasing number of clinical trials using B7-H3 targeting CAR T cell therapies, we examined B7-H3 expression across breast cancer subtypes and in breast cancer brain metastases to assess its potential as an interventional target. B7-H3 expression was investigated using immunohistochemistry on tissue microarrays of three clinical cohorts: (i) unselected primary breast cancers (n = 347); (ii) brain metastatic breast cancers (n = 61) and breast cancer brain metastases (n = 80, including a subset of 53 patient-matched breast and brain metastasis cases); and (iii) mixed brain metastases from a range of primary tumours (n = 137). In primary breast cancers, B7-H3 expression significantly correlated with higher tumour grades and aggressive breast cancer subtypes, as well as poorer 5-year survival outcomes. Subcellular localisation of B7-H3 impacted breast cancer-specific survival, with cytoplasmic staining also correlating with a poorer outcome. Its expression was frequently detected in brain metastases from breast cancers, with up to 90% expressing B7-H3. However, not all brain metastases showed high levels of expression, with those from colorectal and renal tumours showing a low frequency of B7-H3 expression (0/14 and 2/16, respectively). The prevalence of B7-H3 expression in breast cancers and breast cancer brain metastases indicates potential opportunities for B7-H3 targeted therapies in breast cancer management.

## 1. Introduction

Breast cancer brain metastases occur in 10–16% of breast cancer patients and are associated with significant morbidities and mortality [1,2]. The development of breast brain metastases involves intricate interactions between the tumour cells and the brain microenvironment. Presently, there are no biomarkers to predict the spread of a breast or other primary cancer to the brain, which means such metastatic spread can only be detected at the symptomatic presentation of disease. The current treatment for brain metastasis may include neurosurgery, radiotherapy, and/or chemotherapy [3], but depends on clinical factors, including the extent of disease. Systemic chemotherapy is a mainstay; however, achieving efficacious dosing within the brain is challenging. Immunotherapy is emerging as potential targeted therapy in brain metastases [4].

B7 homolog 3 (B7-H3), also known as CD276, belongs to the B7 family of immune regulatory proteins. Located on chromosome 15, B7-H3 exists either as a transmembrane or soluble protein and is expressed in two forms: 2IgB7-H3 (~45–66 kDa) [5] and 4IgB7-H3 (~110 kDa) [6]. Unlike the other members of the B7 family, B7-H3 expression is not limited to professional antigen-presenting cells but is widely expressed in tissues like the heart, liver, spleen, prostate, and thymus, with its mRNA abundant but protein expression restricted, suggesting that post-transcriptional regulatory mechanisms are in place [5]. Initially thought to enhance T cell generation, contrasting findings from subsequent studies have reported its role in downregulating T cell proliferation [7]. B7-H3’s function in immunomodulation remains debatable, purportedly interacting with inflammatory cytokines and activating cytotoxic T cells [8]. However, these findings have also faced contradiction [9] and B7-H3’s upregulation in cancer cells adds further complexity.

B7-H3 is reported to have both pro-tumour [5,10] and anti-tumour [11,12,13] roles across different tumour types. B7-H3’s prognostic capacity has been reported for non-small cell lung cancer, prostate cancer, colorectal cancer, and melanoma [12,14,15,16,17,18,19,20,21,22,23,24,25]. In breast cancer, B7-H3 expression was correlated with increased tumour size and lymphovascular invasion, and *B7-H3* mRNA expression in the primary tumour predicted regional lymph node metastasis [25]. Cong et. al. showed that expression of B7-H3 correlated with a poor prognosis, but did not consider breast cancer subtypes separately [24]. High *B7-H3* mRNA levels in the TGCA data were reported to correlate with poor survival in luminal A and luminal B cancers but not the basal and HER2 subtypes [26].

Therapeutically, B7-H3 has been investigated as a potential target for antibody-mediated ultrasound molecular imaging in breast cancer due to its abundant expression in the breast tumour vasculature [25]. Circulating breast epithelial tumour cells were found to be enriched for B7-H3, and a significant association between the proliferation marker Ki67and B7-H3 expression was found [27]. In TNBC cells, B7-H3 glycosylation by FUT8 was linked to immunosuppression and targeting this axis in a TNBC mouse model increased immune-mediated cell death [28] and improved anti-PDL1 therapy efficacy, offering a new therapeutic avenue for better TNBC outcomes.

Within other tumour streams, many B7-H3 targeting agents are being tested. MGC018, an antibody drug conjugate (ADC) targeting B7-H3, is being tested in ongoing trials as a treatment for six types of advanced solid tumours (NCT03729596, including TNBC) [29] and metastatic prostate cancer (NCT05551117), and MGA271 (enoblituzumab) trials are taking place in paediatric patients with B7-H3-expressing relapsed or refractory solid tumours (NCT02982941). Promising results have been seen in ongoing bispecific T cell engager MGD009 and CAR T cell therapy targeting B7-H3 [30], and there are ongoing studies into radioimmunotherapy agents, such as 131I-omburtamab, which primarily focus on CNS and peritoneal tumours, (NCT04022213, NCT00089245, NCT03275402).

The potential significance of B7-H3 expression in breast cancer is still emerging, and its role in the context of brain metastasis is notably underexplored considering the proliferation of therapeutic agents that may be of use in the BrM setting. Therefore, we present an investigation into the relationship between B7-H3 in breast cancer and breast cancer brain metastasis progression in three patient cohorts, with characterisation of its overall expression and localisation.

## 2. Results

### 2.1. B7-H3 and the Cancer Immune Contexture

To understand B7-H3 (CD267) expression in the broader immune–oncology context, we compared *B7-H3* expression levels to the inferred levels of 26 tumour-infiltrated immune cell types or states in samples across 34 cancer types from The Cancer Genome Atlas (TCGA). In breast cancer—and across most other cancer types—this revealed statistically significant positive associations between *B7-H3* mRNA levels and infiltrated regulatory T cells and macrophages, the latter of which were mostly attributable to non-activated (M0) macrophages (Figure S1). Conversely, *B7-H3* expression inversely correlated with the levels of infiltrated lymphocytes, especially CD8^+^ T cells and memory B cells (each *p* < 0.001), which is largely consistent with correlations in other cancer types (Figure S1). Compared to normal breast tissue, there was an increase of *B7-H3* mRNA expression in breast cancer with comparable expression across subtypes and stages (Figure S2).

**Figure 1:**
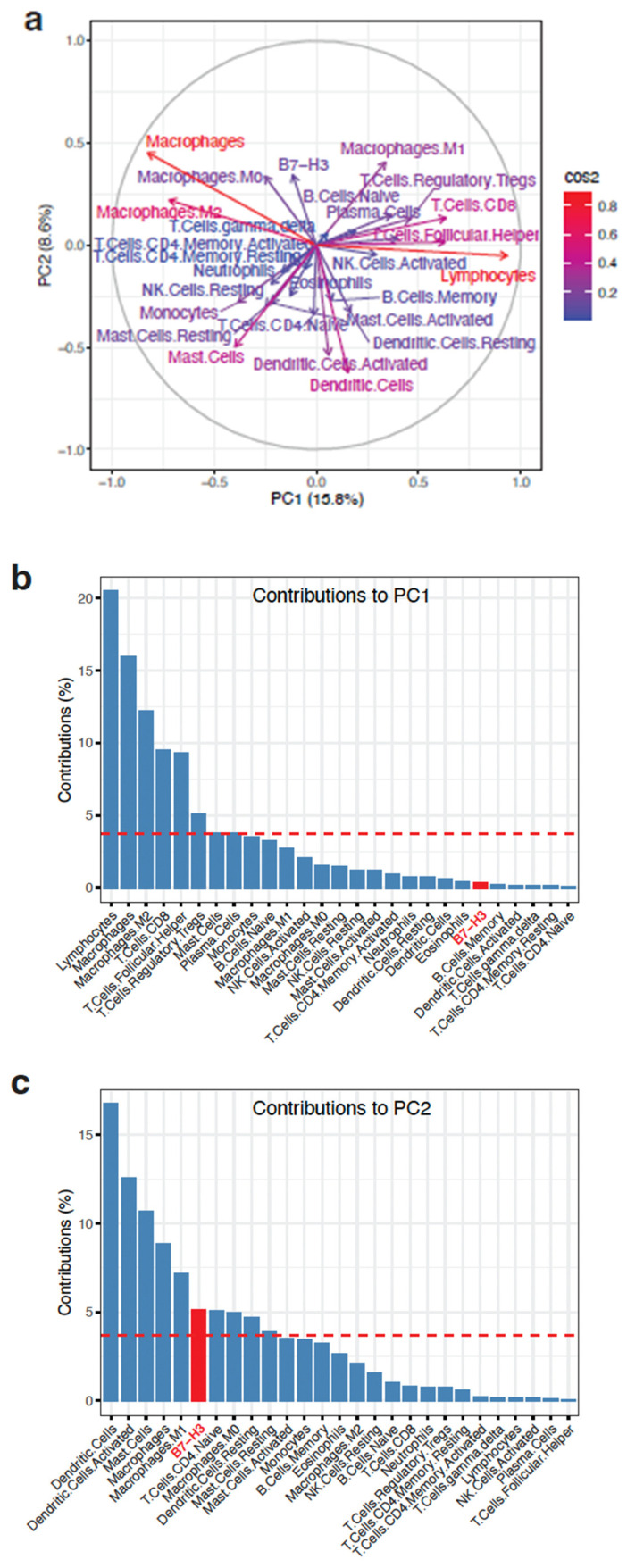
Principal component analysis of *B7-H3* expression and infiltrated immune cell types. (**a**) PCA showing the contributions of the respective infiltrated immune cell types or states and B7-H3 to the first two principal components (PC1 and PC2). Colours of the arrows for each variable are proportional to square cosine (cos2), showing the degree of representation of the variables to PC1 and PC2. (**b**,**c**) Decomposition of PC1 (**b**) and PC2 (**c**) showing the contributions of each of the variables. The dotted horizontal line indicates the contribution level if all variables had uniformly contributed. *B7-H3* is highlighted in red.

To determine the extent to which *B7-H3* expression may contribute to the infiltrated immune cell landscape, we performed principal component analysis (PCA) using the levels of all of the above mentioned infiltrated immune cell types and states, as well as the *B7-H3* expression levels. This revealed variable contributions of each of these factors to the first and second principal components (PC1, PC2), which contributed 15.8 and 8.6% to the variance, respectively (Figure 1a). Decomposition of PC1 and PC2 showed that *B7-H3* contributed above the level of uniform contributions to PC2 but not to PC1 (Figure 1b,c). Notably, macrophages constituted the only variable that contributed above the level of uniform contributions for both PC1 and PC2.

### 2.2. Clinicopathological Analysis of B7-H3 in Breast Cancer Subtypes

**Figure 2:**
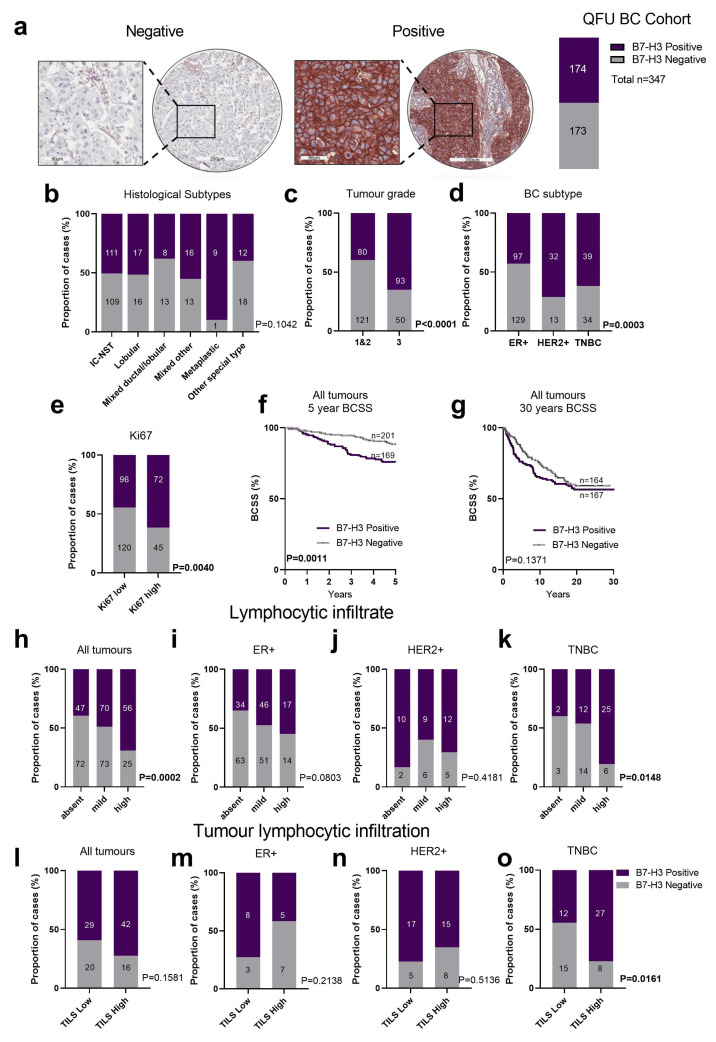
B7-H3 expression in breast tumours in the QFU cohort. (**a**) Representative immunohistochemistry images of breast tumour cores scored as negative (left image) or positive (right image) for B7-H3 expression with 50% of the breast tumours in the QFU cohort stained positive for B7-H3 (n = 173 of 343). (**b**) Chi-square analysis of the proportion of breast cancer histological subtypes with negative or positive B7-H3 expression. (**c**) Chi-square analysis of the proportion of breast cancer cases with positive and negative B7-H3 expression compared across tumour grades. (**d**) Chi-square analysis of the proportion of breast cancer subtypes (ER+, HER2+, and TNBC) with negative and positive B7-H3 expression. (**e**) Chi-square analysis of B7-H3 expression in Ki67-low and Ki-67-high cases across all breast cancer subtypes. (**f**,**g**) Kaplan–Meier (KM) analysis of B7-H3 expression and 5-year (**f**) and 30-year BCSS (**g**). (**h**) Chi-square analysis of lymphocytic infiltration in all tumour subtypes, and in ER+ only (**i**), HER2+ only (**j**), and TNBC only (**k**). (**l**) Chi-square analysis of tumour infiltrating lymphocytes (TILS) in all tumour subtypes, and: ER+ only (**m**), HER2+ only (**n**), and TNBC only (**o**). BC, breast cancer; BCSS, breast cancer specific survival; ER+, oestrogen receptor; HER2+, human epidermal growth factor receptor 2; TILs, tumour infiltrating lymphocytes; TNBC, triple negative breast cancer. Scale bar: core = 200 µm; inset 60 µm.

B7-H3 expression in primary breast cancers was investigated using immunohistochemistry (IHC) in the QFU cohort [31], which comprises 347 unselected breast cancer patients. In the QFU cohort, 50% of the cancers showed some degree of tumour cell specific expression (>1% tumour cells) of B7-H3 (Figure 2a), reaching 90% of cases in the metaplastic breast cancer subtype (n = 10; Figure 2b). Analysing B7-H3 expression in relation to clinicopathology features (Table 1) revealed that Grade 3 tumours more highly expressed B7-H3 compared to Grade 1 and 2 tumours (Figure 2c, *p* < 0.0001, Chi-square). HER2+ and TNBC subtypes exhibited a significant increase in B7-H3 expression compared to the ER+ breast cancer subtype (*p* = 0.0003, Chi-square, Figure 2d). B7-H3 positivity was associated with highly proliferative breast tumours, with a significant association between B7-H3 and the Ki67 proliferation marker (Figure 2e, *p* = 0.0040, Chi-square). This association was driven solely by TNBC tumours (Figure S3).

We then assessed the relationship between B7-H3 expression and survival, and found that B7-H3 expression was significantly associated with poorer 5-year breast cancer specific survival (BCSS) in the unselected breast cancer cohort (Figure 2f, *p* = 0.0011, log rank). However, over the longer term, the absence of B7-H3 expression lost its prognostic advantage (Figure 2g). B7-H3 expression did not demonstrate a significant association with survival across either grade or breast cancer subtype (ER+, HER2+, and TNBC) (Figure S4A). Within the HER2+ group, B7-H3 positivity trended towards worse BCSS, and in TNBC the opposite was seen, with B7-H3 expression associated with an improved prognosis; however, the case numbers were low (Figure S4B). In a Cox Proportional Multivariate analysis (Table S1), B7-H3 expression did not contribute any significant independent prognostic power. Indeed, HER2 status was the most powerful over 5 years (*p* = 0.002, HR = 3.9, CI 1.62–9.4) and 30 years’ follow-up of breast cancer specific survival (*p* = 0.0006, HR = 2.88, CI 1.58–5.26), which is to be expected given the historic nature of our cohort, which pre-dates anti-HER2 therapies.

We next investigated the association of B7-H3 expression with immune features within our cohort. We scored immune features in two ways: (i) the extent of infiltration of lymphocytes across the tumour (classified as absent, mild, or high); and (ii) according to the International Working Group [32], which defines TILs as being within the stroma of the tumour, and tumours are classified as “TILs Low” or “TILs High”, reflecting <20% or >20% lymphocytic infiltration, respectively. For the three-tier classification, B7-H3 positivity was significantly associated with high lymphocytic infiltration in unselected breast cancers (Figure 2h, *p* = 0.0002, Chi-square) and TNBC (Figure 2k, *p* = 0.0148, Chi-square), but not within ER+ (Figure 2i) and HER2+ cases (Figure 2j). In the two-tier classification, B7-H3 positivity was more frequent, but not significantly associated with the TILs High group in unselected cases (Figure 2l), and was significantly associated with TILs High in the TNBC subtype (Figure 2l–o, *p* = 0.0161, Chi-square).

### 2.3. Characterisation of B7-H3 Subcellular Localisation in Breast Cancer

**Figure 3:**
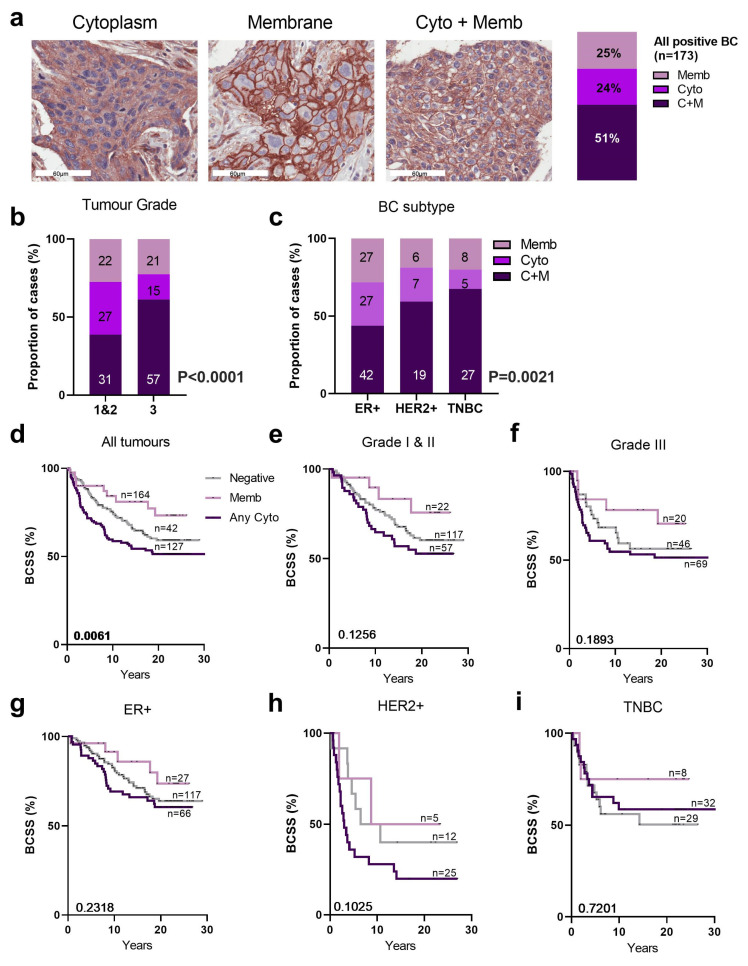
Subcellular localisation of B7-H3 in QFU cohort. (**a**) Representative immunohistochemistry images highlighting membrane (memb), cytoplasmic (cyto), and cytoplasmic + membrane (C+M) B7-H3 expression. B7-H3 expression localised in membrane, cytoplasm, or both (C+M) (24.3%, 24.9%, and 50.9% respectively). (**b**) Chi-square analysis of the proportion of breast cancer cases with membrane (memb), cytoplasmic (cyto), and cytoplasmic + membrane (C+M) B7-H3 expression by tumour grade and (**c**) breast cancer subtypes (ER+, HER2+, TNBC; right). KM analysis of (**d**) all tumours, (**e**) tumour grade 1 and 2, and (**f**) Grade 3, stratified by B7-H3 membrane vs. all cytoplasmic expression and BCSS. KM analysis of breast cancer subtype ER+ (**g**), HER2+ (**h**), and TNBC (**i**) stratified by B7-H3 membrane vs. cytoplasmic expression and BCSS. BC, breast cancer; BCSS, breast cancer specific survival; cyto, cytoplasmic; C+M, cytoplasmic + membrane; ER+, oestrogen receptor positive; HER2+, human epidermal growth factor receptor 2; memb, membrane; TNBC, triple negative breast cancer. Scale bar = 60 µm.

B7-H3 is expressed in the membrane and cytoplasm, and occasionally in the nucleus [33], which prompted us to investigate B7-H3 localisation. We observed a range of patterns of B7-H3 expression, with membrane localisation (Memb, 25%), cytoplasmic (Cyto, 24%), or both (C+M, 51%), as shown in Figure 3a. There was no nuclear localisation observed. There was an increase of C+M B7-H3 localisation in Grade 3 tumours compared to Grade 1 and 2 (Figure 3b, *p* < 0.001, Chi-square). This was also true among the HER2+ and TNBC cases, which exhibited a higher frequency of C+M localisation of B7-H3 compared to ER+ cases (Figure 3c, *p* = 0.0021, Chi-square).

The impact of B7-H3 subcellular localisation on BCSS showed breast cancers with any cytoplasmic staining (both cytoplasmic only, and C+M) had the poorest BCSS, while membrane-only staining was associated with the best prognosis (Figure 3d; *p* = 0.0061, log rank). No significant associations with survival were observed when we stratified the cohort by tumour grade (Figure 3e,f) or subtype (Figure 3g–i). In a Cox proportional multivariate analysis (Table S2), B7-H3 subcellular localisation did not contribute independent significant prognostic power. Indeed, HER2 status was the most powerful over 5 years (*p* = 0.002, HR = 4.04, CI 1.66–9.8) and with 30 years of breast cancer specific survival (*p* = 0.0006, HR = 2.88, CI 1.57–5.29).

### 2.4. Clinicopathological Analysis of B7-H3 Expression in Breast Cancer Brain Metastases

**Figure 4:**
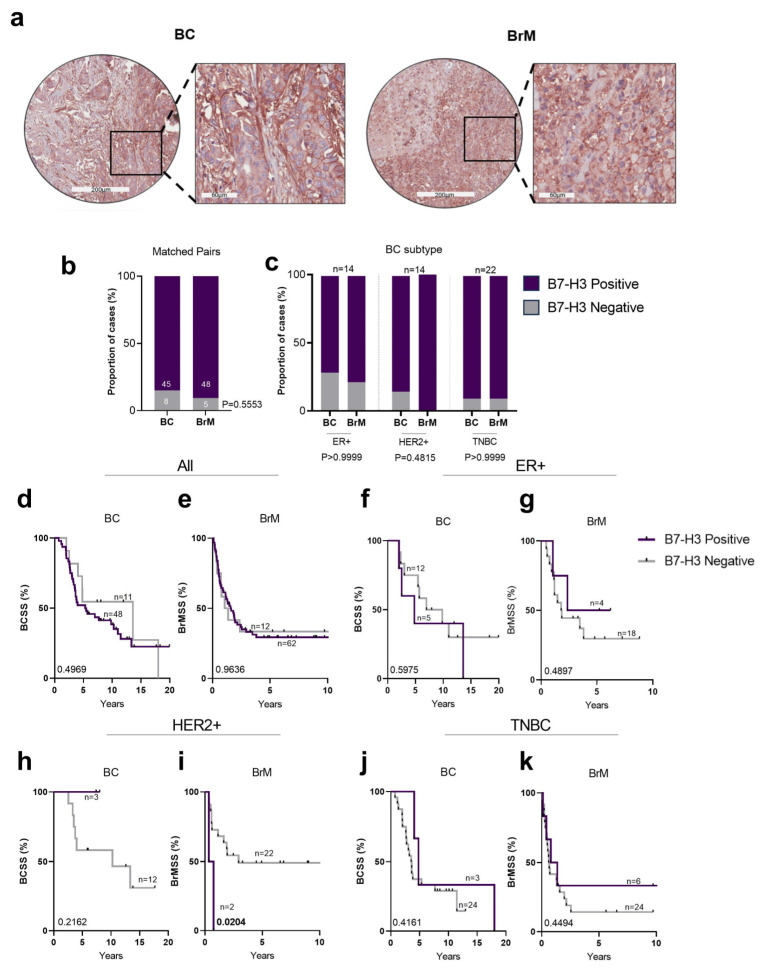
B7-H3 staining in breast tumours in the QBBM cohort. (**a**) Representative immunohistochemistry images of matched breast tumour and brain metastasis for B7-H3 staining. (**b**) Chi-square analysis of the proportion of matched breast cancer and BrM cases with positive and negative B7-H3 expression. (**c**) Chi-square analysis of proportions of matched breast cancer and BrM cases stratified by B7-H3 staining compared across BC subtypes (ER+, HER2+, and TNBC). (**d**–**k**) KM analysis of B7-H3 expression and BCSS cases compared across all tumours (**d**), ER+ (**f**), HER2+ (**h**), and TNBC (**j**). KM analysis of B7-H3 expression and BrMSS cases compared across all tumours (**e**), ER+ (**g**), HER2+ (**i**), and TNBC (**k**). Note the X axis changes between breast cancer and BrM. BC, breast cancer; BrM, brain metastasis; HER2+, human epidermal growth factor receptor 2; ER+, oestrogen receptor; KM, Kaplan–Meier; TNBC, triple negative breast cancer. Scale bar: core = 200 µm; inset 60 µm.

Next, we explored B7-H3 expression in the Queensland breast cancer-brain metastasis cohort (QBBM), which consists of 61 breast cancer cases and 80 BrM tumours, including 53 matched breast cancer-BrM pairs (Figure 4a). Statistical associations between B7-H3 expression and clinicopathological variables are shown in Table 2.

Overall, we observed more expression in BrMs than BCs in the QBBM cohort (90% vs. 84%; Figure 4b). Comparative analysis of B7-H3 expression between matched breast cancer and BrM tumours revealed that ER+ cases showed the lowest number of cases with B7-H3 expression (Figure 4c). In the HER2+ subtype, 90% of brain metastatic primary tumours showed positivity for B7-H3, with this increasing to 100% positivity in the BrM tumours. Meanwhile, 90% of brain metastatic TNBCs were positive, as were the BrMs arising from TNBC (Figure 4c). Considering B7-H3 was almost uniformly highly expressed within the cohort, it is not surprising that no significant association with BCSS was found (Figure 4d,f,h,j). There was a trend towards a poorer BrMSS between breast cancer and BrM tumours; however, it was not significant, likely due to the unequal numbers of patients in each group (Figure 4e,g,i,k).

### 2.5. Characterisation of B7-H3 Subcellular Localisation in Breast Brain Metastases

**Figure 5:**
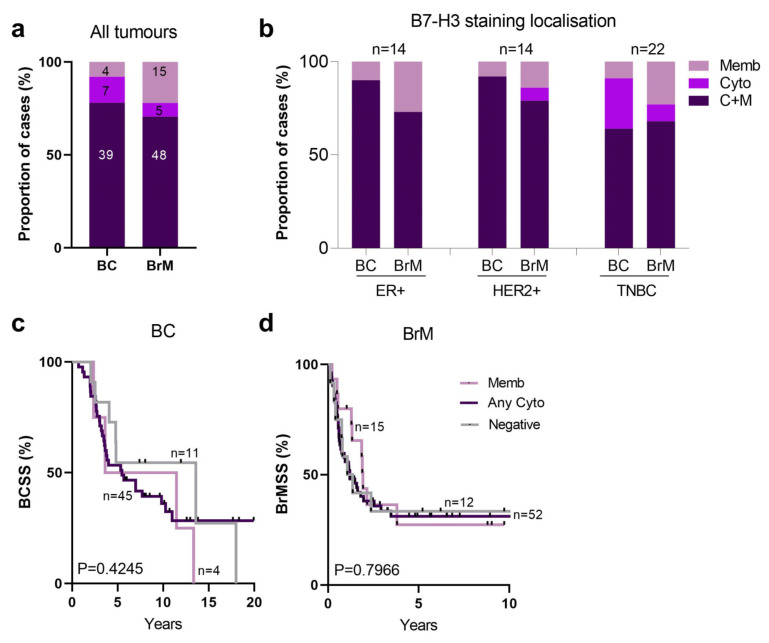
Subcellular localisation of B7-H3 in breast cancer-BrM tumours. (**a**) Chi-square analysis of proportions of BC and BrM cases stratified by B7-H3 subcellular localisation. (**b**) Chi-square analysis of proportions of matched BC and BrM cases for B7-H3 subcellular localisation compared across breast cancer subtypes (ER+, HER2+, TNBC). (**c**) KM analysis of B7-H3 subcellular localisation and BCSS in BC. (**d**) KM analysis of B7-H3 subcellular localisation and BrMSS in BrM tumours. BC, breast cancer; BCSS, breast cancer specific survival; BrMSS, brain metastasis specific survival; ER+, oestrogen receptor; HER2+, human epidermal growth factor receptor 2; KM, Kaplan–Meier; TNBC, triple negative breast cancer.

In the BrM cohort, the most prevalent B7-H3 staining pattern was C+M, with similar proportions across both the primaries (78%) and brain metastases (70%) (Figure 5a). For the matched cases stratified according to breast cancer subtype (ER+, HER2+, TNBC), we observed an equal proportion of cases with C+M B7-H3 expression and that membrane-only expression increased in the BrM cases in each subtype (Figure 5b). BC and BrM cases stratified by B7-H3 localisation did not show a significant association with survival for either BCSS (Figure 5c) or BrMSS (Figure 5d).

### 2.6. Characterisation of B7-H3 Expression Varies in Brain Metastases Derived from a Range of Primary Tumours

**Figure 6:**
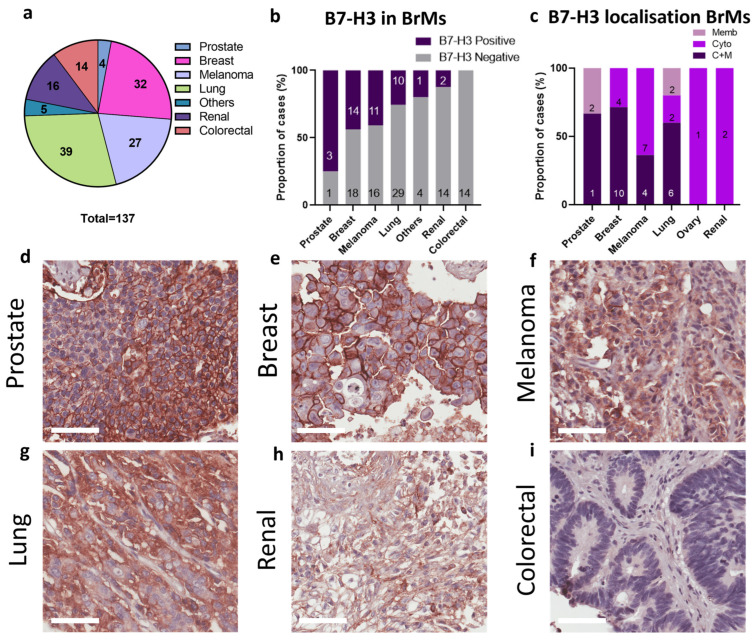
B7-H3 expression in an independent brain metastasis cohort. (**a**) The mixed brain metastasis cohort was comprised of samples from 137 BrMs derived from different primary tumours. (**b**) The proportion of BrM cases with positive and negative B7-H3 expression across the cohort; ‘others’ consists of ovary, ACC, and NET due to low numbers. (**c**) B7-H3 subcellular localisation across BrM cases. (**d**–**i**) Representative B7-H3 immunohistochemistry images from the mixed brain metastasis cohort: prostate (**d**), breast (**e**), melanoma (**f**), lung (**g**), renal (**h**), and colorectal cancer (**i**). ACC, adenoid cystic carcinoma; BrM, brain metastasis; NET, neuroendocrine tumour. Scale bar: 60 µm.

We subsequently investigated B7-H3 expression in an independent brain metastasis cohort, with metastases originating from a variety of primary tumours: prostate, breast, melanoma, lung, renal, colorectal cancer, ovary, neuroendocrine, and adenoid cystic carcinoma; Figure 6a. In this independent cohort, we observed B7-H3 expression in 44% of breast-derived BrMs (Figure 6b), lower than that of our QBBM cohort (90%); however, we have no clinicopathology data for these heavily pre-treated samples and cannot further investigate. We also observed B7-H3 expression in BrMs from prostate, lung, and melanoma primaries but no B7-H3 expression was found in colorectal BrMs (Figure 6c–h), and a low frequency of staining was recorded for renal carcinoma-derived BrMs. As for the QBBM cohort, the most prevalent B7-H3 staining pattern for the independent brain metastasis cohort was C+M in breast to brain metastases (71%; Figure 6c). This was also the case for prostate (C+M, 67%), melanoma (C+M, 36%), and lung (C+M, 60%) (Figure 6c).

## 3. Discussion

As B7-H3 therapies approach the clinic [34], we assessed whether different breast cancer subtypes and their brain metastases express this immuno-oncology target. In our breast cancer cohort, B7-H3 was expressed in 50% of cases, particularly in breast cancers with aggressive phenotypes such as grade 3, HER2+, and TNBC tumours. Further, there was a significant positive association between B7-H3 positivity and high Ki67 expression, suggesting a link between B7-H3 expression and tumour cell proliferation. Although no significant association was found between B7-H3 and Ki67 in ER+ and HER2+ cases, TNBC cases showed a significant enrichment of B7-H3 expression in Ki67-high tumours. In our cohort, B7-H3 expression was significantly associated with poor 5-year BCSS. Similarly, B7-H3 expression in hepatocellular carcinoma patients was associated with early recurrence within 1 year and 2-year overall survival [35], and with early tumour-node-metastasis stage in pancreatic cancer [36].

We studied the association between B7-H3, lymphocytic infiltrate and stromal TILs to gain insight into the potential immunomodulatory role of B7-H3 in breast cancer. In our cohort, B7-H3 protein expression was positively correlated with a high lymphocytic infiltrate and stromal TILs in the breast cancers, particularly in the TNBC cases. Though lymphocytic cells in the tumour do not necessarily correlate with an active anti-tumour immune response [37], lymphocytes infiltrating the tumour stroma or the tumour itself in TNBC are an independent factor for favourable survival outcomes [38]. From the TCGA dataset analysis, we noted that *B7-H3* mRNA expression was positively correlated with regulatory T cells and negatively associated with CD8+ T cells in breast cancer. As regulatory T cells can suppress the anti-tumour immune response [39] and CD8+ T cells are effectors of antitumour immune responses [40], the data support that B7-H3 expression may contribute to an immune-suppressive microenvironment, aligning with the findings of Huang et al. [28]. In other cancers, B7-H3 high expression has been shown to be positively associated with the immunosuppressive FOXP3+ regulatory T cells in renal cell carcinoma [41] and non-small cell lung cancer [42]. Therefore, a more detailed assessment of B7-H3 and the immune context, including markers of TILS such as FOXP3, CD3, CD4, CD8, and CD56, is needed.

The cross-talk between B7-H3 and immunotherapy targets such as PD-1 is also an important consideration for the targetable potential of B7-H3. In NSCLCs, B7-H3 is associated with non-responsiveness to anti-PD-1 immunotherapy, whereas targeting B7-H3 along with anti-PD-L1 showed significant anti-tumour efficacy [43]. The clinical efficacy of this approach was recently shown in a phase I/II trial for HNSCC [NCT02475213] and NSCLC patients [44], where the combination of B7-H3 and PD-1 blockade was shown to be a safe and effective anti-tumour treatment option.

B7-H3 has been reported to be localised in the cytoplasm and membrane, with occasional expression in the nucleus [33]. In our study, B7-H3 was localised either exclusively in the cytoplasm or membrane, or was found in both the membrane and cytoplasm, but no nuclear staining was observed. The cytoplasmic and membranous staining of B7-H3 was significantly enriched in grade 3 tumours, as well as in HER2+ and TNBC tumours. Membrane B7-H3 expression was found to be significantly associated with favourable BCSS. Although the link between B7-H3 localisation and survival outcomes has not previously been reported for breast cancer, in renal cell carcinoma, cytoplasmic B7-H3 was similarly found to be significantly associated with worse disease-specific survival as well as lymph node invasion [45].

Aggressive breast cancers have a high tendency to spread to the brain [46]. In contrast to the primary breast cancer cohort (50% positivity), B7-H3 expression was observed in 80% of brain metastatic breast cancers and up to 90% of their respective BrM tumours. There were no significant clinicopathological variable associations due to the almost uniformly positive staining. The increased expression of B7-H3 in brain metastatic primary tumours relative to unselected primary breast tumours suggests a role in brain metastasis. However, the independent brain metastasis cohort revealed that not all brain metastases have high B7-H3 expression, with colorectal cancer-derived BrM completely lacking staining, and only 2/16 of renal cancer BrMs showed B7-H3 expression. Indeed, in this independent cohort, only 44% of BC-BrMs showed B7-H3 expression compared to 90% in our QBBM cohort. Given that brain metastasis patients had undergone diverse therapeutic treatments, these findings are perhaps not unexpected. However, they do warrant further study in a larger cohort of samples with detailed treatment histories, particularly in light of the nuclear staining reported to be associated with tumour progression in colorectal samples [11]. Although B7-H3 is not well studied in BrMs, in the context of primary brain tumours, B7-H3 is implicated in immune evasion via dampening of the immune response and the promotion of angiogenesis via mediating neovascular endothelial cell proliferation and capillary formation [46,47]. Inhibition of B7-H3 in a preclinical breast BrM model was shown to stimulate CD8 production, reduce metastatic growth, and increase survival [48]. In addition, studies in brain tumours revealed the role of B7-H3 in regulating cancer stemness and metabolism [49]. As the pathogenesis of BrM involves brain metastatic cells undergoing cycles of dormancy, neural microenvironment adaptation, and brain invasion [50,51], it is possible that B7-H3 may be involved in these mechanisms for cells originating in the breast.

B7-H3 targeting in cancer by drug conjugates and CAR T cell therapy has been shown to facilitate a strong anti-tumour response in paediatric and solid tumours [52,53]. Our data show B7-H3 to be expressed in both BC and BrM tumours, thus investigating the efficacy of targeting B7-H3 using CAR T cells, or by using antibody-drug-conjugates, will be an important next step in understanding the therapeutic potential of B7-H3 in breast cancer and breast cancer brain metastases. Given its low expression in non-tumour cells, B7-H3 is a promising target with minimal off-target effects.

A limitation is that we did not explore the significance of B7-H3 isoforms, of which there are several. The antibody used in this study detects both the 2Ig-B7-H3 and 4Ig-B7-H3 forms but not the soluble B7-H3 isoform. Although 4Ig-B7-H3 is the more prominent form of B7-H3 found in malignant cells [54], mRNA expression of *2Ig-B7-H3* was shown to be associated with worse overall survival in acute myeloid leukaemia [55]. Distinguishing between the B7-H3 isoforms would give more detailed insights into its association with breast cancer and brain metastases in the future.

In summary, B7-H3 expression was found to be a prognostic marker of early survival outcomes of breast cancer and was shown to be highly expressed in brain metastatic breast cancer and BrMs. The high prevalence of B7-H3 expression in breast cancer brain metastases makes them potential candidates for B7-H3 targeted therapies.

## 4. Materials and Methods

### 4.1. Clinical Cohorts

This study received ethical approval from the Royal Brisbane Women’s Hospital (RBWH) (2005/022) and The University of Queensland (2005000785) for the use of clinical data and samples from patients.

Three clinical cohorts were studied: (1) the Queensland Follow-Up (QFU) cohort, which is comprised of formalin-fixed paraffin-embedded (FFPE) breast tumour samples from 347 patients that underwent breast tumour resection at the Royal Brisbane Women’s Hospital between 1987 to 1994, along with their long-term clinical follow-up information (median 13.5 years; range: 0.2 to 42 years) [31]. (2) Queensland Breast cancer-Brain Metastasis (QBBM) cohort comprised of brain-metastatic breast cancer patients (n = 61) and brain metastasis (BrM) samples (n = 80), including 53 matched breast cancer-BrM pairs, treated between the years 2000 and 2018 [56]. (3) A mixed brain metastasis cohort, consisting of 137 brain metastases derived from different primary tumours: prostate, breast, melanoma, lung, others (neuroendocrine, adenoid cystic, ovary), renal, and colorectal cancer [57].

Tumours were sampled in tissue microarrays (TMA) as 0.6 mm (QFU) or 1 mm (QBBM) cores for immunohistochemical (IHC) analysis. Pathology reports, clinical diagnostic information, and survival data were obtained from Pathology Queensland, Queensland Health, and the Queensland Cancer registry. The clinicopathological information was curated; however, complete treatment information is limited in these cohorts due to the historical nature of the dataset. Survival analysis was assessed as breast cancer-specific survival (BCSS; calculated from the time of breast cancer diagnosis to the last follow up), and brain metastasis specific survival (BrMSS; calculated from the time of BrM resection surgery to the last known follow up).

### 4.2. Immunohistochemistry

Antigen retrieval was performed on 4 µm sections with heat-induced epitope retrieval in a decloaking chamber (Nexgen, Biocare Medical, Concord, CA, USA) with sodium citrate buffer (0.01 M, pH 6.0) at 110 °C for 20 min. The reagents from the MACH1 Universal HRP detection Kit (Biocare Medical, LLC, Concord, CA, USA, #M1U539 L10) was used for immunohistochemical detection. MACH1 sniper blocking reagent was used to block non-specific staining for 30 min. Primary antibodies against B7-H3 (1:200; Cell Signaling, #D9M2L/#14058, Beverly, MA, USA) and Ki-76 (1:100; Dako, #M7240, Santa Clara, CA, USA) were diluted in Biocare Da Vinci Green Diluent (Biocare Medical, #PD900, Concord, CA, USA) and incubated overnight at room temperature. The slides were treated with MACH1 secondary antibody conjugated with MACH1 Horse-Radish Peroxidase polymer for 30 min at room temperature. MACH1 diaminobenzidine substrate was applied for 5 min and the slides were counterstained with hematoxylin.

### 4.3. Biomarker Scoring and Analysis

The scoring was performed by a pathologist (AS). A positive B7-H3 stain was defined as >1% of cells displaying unequivocal staining, whereas a negative finding was defined as <1% positivity as described previously [56]. Additionally, the subcellular localisation pattern of B7-H3 protein expression was documented as either cytoplasmic-only, membrane-only, or cytoplasmic + membrane (C+M); no nuclear staining was observed. For the survival analysis, those cases featuring any cytoplasmic staining (i.e., cytoplasmic-only or cytoplasmic + membrane) were grouped together.

### 4.4. Tumour Infiltrating Lymphocytes (TILs) and Lymphocytic Infiltration Scoring

Tumour immune cell infiltration was scored on hematoxylin and eosin-stained whole sections within the boundary of the tumour by pathologists (AS, SRL). Using the International Immuno-Oncology Biomarker Working Group [32] guidelines, tumour infiltrating lymphocytes were quantitated by the area occupied by mononuclear inflammatory cells (lymphocytes and plasma cells) over the total stromal area. TILs within the borders, invasive edges, and stroma of the tumours were included in the evaluation.

### 4.5. Estimation of Immune Cell Infiltration Levels in Cancers

Batch effects-normalized mRNA levels ($log_{2}\left(normalized\;counts+1\right)$ values) using Illumina HiSeq RNASeqV2 were obtained from the pan-cancer dataset of The Cancer Genome Atlas (TCGA) across 33 cancer types [58]. For non-small cell lung cancers (NSCLC), lung adenocarcinoma (LUAD) and lung squamous cell carcinoma (LUSC) samples were combined. The levels of 26 tumour-infiltrated immune cell types or states were estimated in these samples using the tumour immune estimation resource, as previously described [59]. These levels were compared to expression levels of *B7-H3* in the same samples using Spearman correlations. Spearman *R* and *p* values were determined in *R* (version 4.3.1; The R Project for Statistical Computing) [60] and heatmaps were created using the *tidyverse* package [61].

### 4.6. Principal Component Analysis

For Principal Component Analysis (PCA), levels of all infiltrated immune cell types and states (determined as described above), as well as the mRNA expression levels of B7-H3, were included. PCA was performed using *R* (version 4.3.2; 31 October 2023) and the *R* packages *factoextra* (version 1.0.7; https://CRAN.R-project.org/package=factoextra, accessed on 19 March 2024) and *ggplot2* (version 3.5.0; https://ggplot2.tidyverse.org, accessed on 19 March 2024)

### 4.7. Statistics

Raw data from each experiment was analysed using GraphPad Prism (v9.0). For the multivariate survival analysis, we utilised the Survivalanalysis package in *R* (version 4.3.2, accessed on 21 March 2024) [62]. The statistical tests performed for each experiment as well as the *p* value are indicated in the respective figure legends. A *p*-value of <0.05 was used as the cut-off for statistically significant findings.

## Figures and Tables

**Table 1:** Clinicopathological characteristics of breast cancers stratified by B7-H3 expression.

Characteristic	Negative (n (%))	Positive (n (%))	*p* Value	Statistical Test
Total (n = 347)	173 (50%)	174 (50%)		
Age of Diagnosis				
>40	146 (49%)	148 (53%)	0.5898	Fisher’s exact test
<40	15 (44%)	19 (56%)		
Tumour Size (cm)				
2	61 57%)	45 (43%)	0.0642	Chi-square
2–5	45 (42%)	61 (53%)		
>5	9 (41%)	13 (5%)		
Tumour Grade				
1	32 (70%)	14 (30%)	**<0.0001**	Chi-square
2	89 (57%)	66 (43%)		
3	50 (35%)	93 (65%)		
Lymph Node				
Negative	35 (46%)	42 (54%)	0.3299	Fisher’s exact test
Positive	29 (37%)	49 (63%)		
Subtype				
ER+	129 (57%)	97 (43%)	**0.0003**	Chi-square
HER2+	13 (29%)	32 (71%)		
TNBC	24 (38%)	39 (62%)		

**Table 2:** Clinicopathological characteristics of BC-BrM from the QBBM cohort stratified by B7-H3 expression.

	Characteristic	Negative (n (%))	Positive (n (%))	*p* Value	Statistical Test
Primary breast (n = 61)	Subtype				
	ER+	5 (28%)	13 (72%)	0.529	Chi-square
	HER2+	3 (20%)	12 (80%)		
	TNBC	3 (11%)	25 (89%)		
	Total	11 (18%)	50 (82%)		
	Tumour Grade				
	1/2	3 (20%)	12 (80%)	0.993	Chi-square
	3	8 (19%)	35 (81%)		
	Total	11 (19%)	47 (81%)		
	Time to Neurosurgery (Median Years)	2.910335	2.514716	0.597	Welch’s *t* test
				**0.047**	F test
Brain Metastasis (n = 80)	B7-H3 staining	12 (15%)	68 (85%)	0.6518	* Fisher’s exact test

* Compared to total primary breast cancers.

## Data Availability

The data presented in this study are available in this article and Supplementary Materials.

## Supplementary Materials

The following supporting information can be downloaded at: https://www.mdpi.com/article/10.3390/ijms25073976/s1.
