# Supplementary material for: B7-H3 Expression in Breast Cancer and Brain Metastasis
Source: Int J Mol Sci. 2024 Apr 3;25(7):3976. doi: 10.3390/ijms25073976 (PMC11012592; doi:10.3390/ijms25073976)
Supplement: Supplementary file 1 [file ijms-25-03976-s001.zip › Figures S1-4.pptx]

## Slide 1
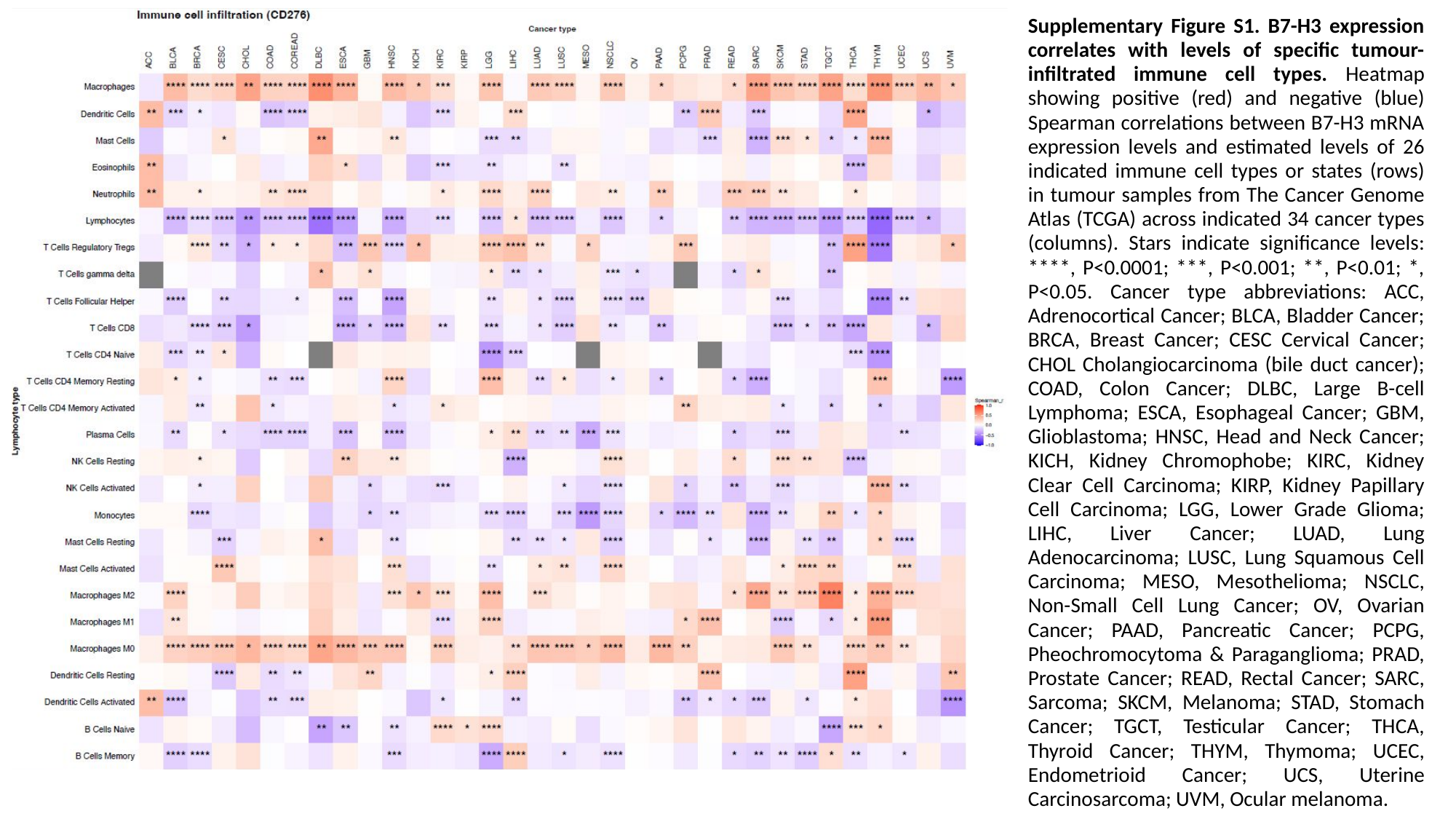

Supplementary Figure S1. B7-H3 expression correlates with levels of specific tumour-infiltrated immune cell types. Heatmap showing positive (red) and negative (blue) Spearman correlations between B7-H3 mRNA expression levels and estimated levels of 26 indicated immune cell types or states (rows) in tumour samples from The Cancer Genome Atlas (TCGA) across indicated 34 cancer types (columns). Stars indicate significance levels: ****, P<0.0001; ***, P<0.001; **, P<0.01; *, P<0.05. Cancer type abbreviations: ACC, Adrenocortical Cancer; BLCA, Bladder Cancer; BRCA, Breast Cancer; CESC Cervical Cancer; CHOL Cholangiocarcinoma (bile duct cancer); COAD, Colon Cancer; DLBC, Large B-cell Lymphoma; ESCA, Esophageal Cancer; GBM, Glioblastoma; HNSC, Head and Neck Cancer; KICH, Kidney Chromophobe; KIRC, Kidney Clear Cell Carcinoma; KIRP, Kidney Papillary Cell Carcinoma; LGG, Lower Grade Glioma; LIHC, Liver Cancer; LUAD, Lung Adenocarcinoma; LUSC, Lung Squamous Cell Carcinoma; MESO, Mesothelioma; NSCLC, Non-Small Cell Lung Cancer; OV, Ovarian Cancer; PAAD, Pancreatic Cancer; PCPG, Pheochromocytoma & Paraganglioma; PRAD, Prostate Cancer; READ, Rectal Cancer; SARC, Sarcoma; SKCM, Melanoma; STAD, Stomach Cancer; TGCT, Testicular Cancer; THCA, Thyroid Cancer; THYM, Thymoma; UCEC, Endometrioid Cancer; UCS, Uterine Carcinosarcoma; UVM, Ocular melanoma.

## Slide 2
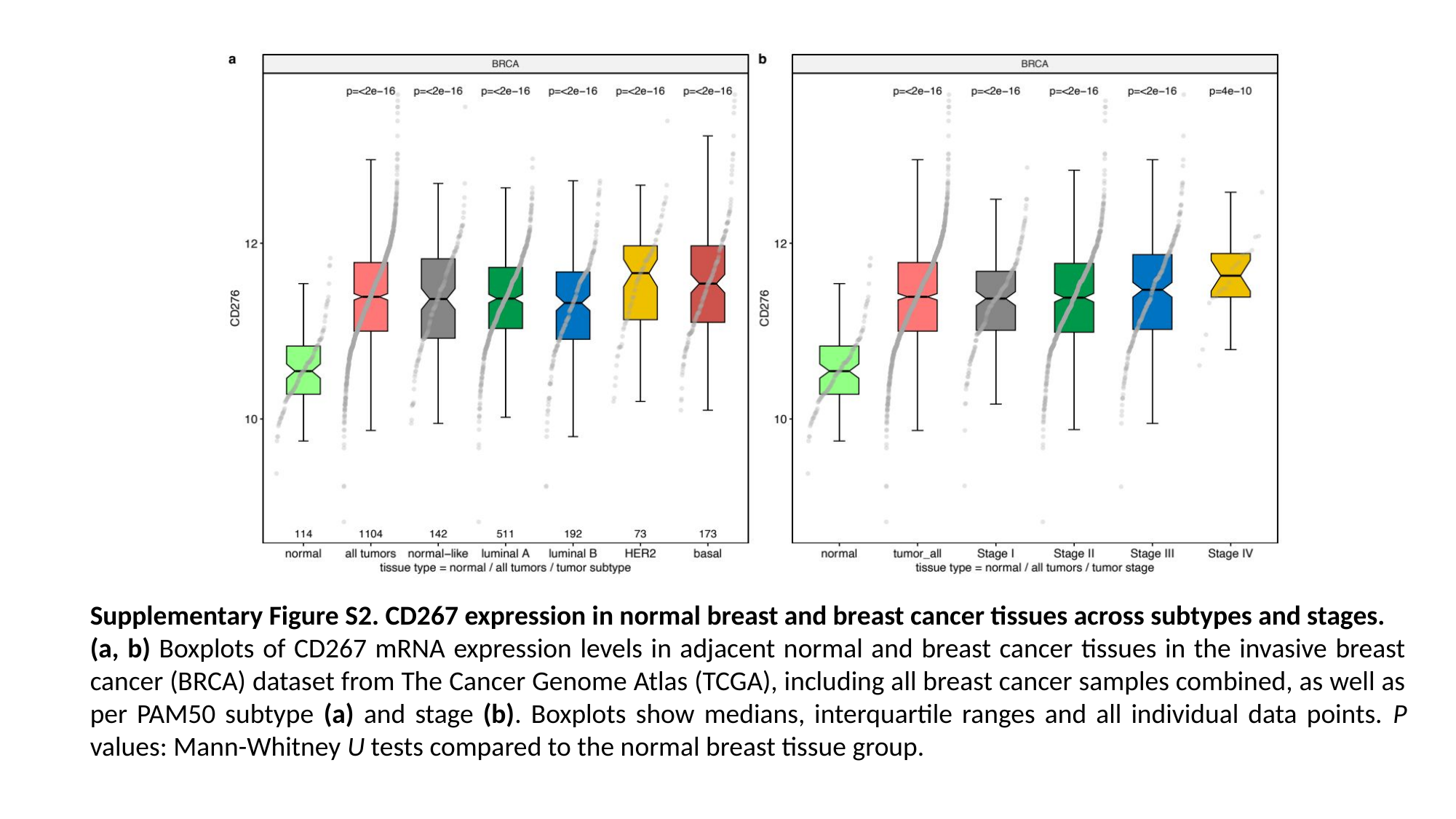

Supplementary Figure S2. CD267 expression in normal breast and breast cancer tissues across subtypes and stages.
(a, b) Boxplots of CD267 mRNA expression levels in adjacent normal and breast cancer tissues in the invasive breast cancer (BRCA) dataset from The Cancer Genome Atlas (TCGA), including all breast cancer samples combined, as well as per PAM50 subtype (a) and stage (b). Boxplots show medians, interquartile ranges and all individual data points. P values: Mann-Whitney U tests compared to the normal breast tissue group.

## Slide 3
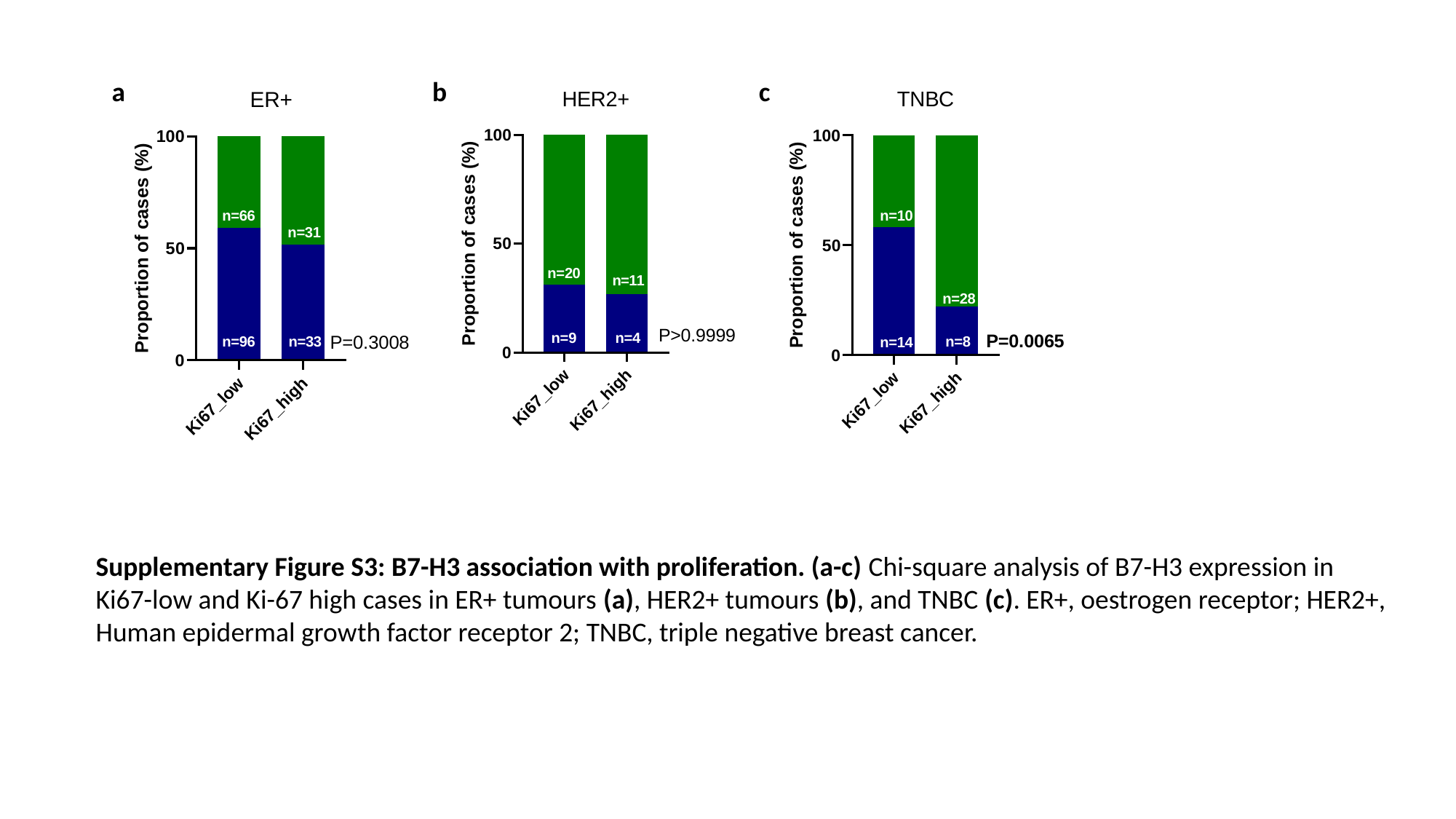

a
b
c
Supplementary Figure S3: B7-H3 association with proliferation. (a-c) Chi-square analysis of B7-H3 expression in Ki67-low and Ki-67 high cases in ER+ tumours (a), HER2+ tumours (b), and TNBC (c). ER+, oestrogen receptor; HER2+, Human epidermal growth factor receptor 2; TNBC, triple negative breast cancer.

## Slide 4
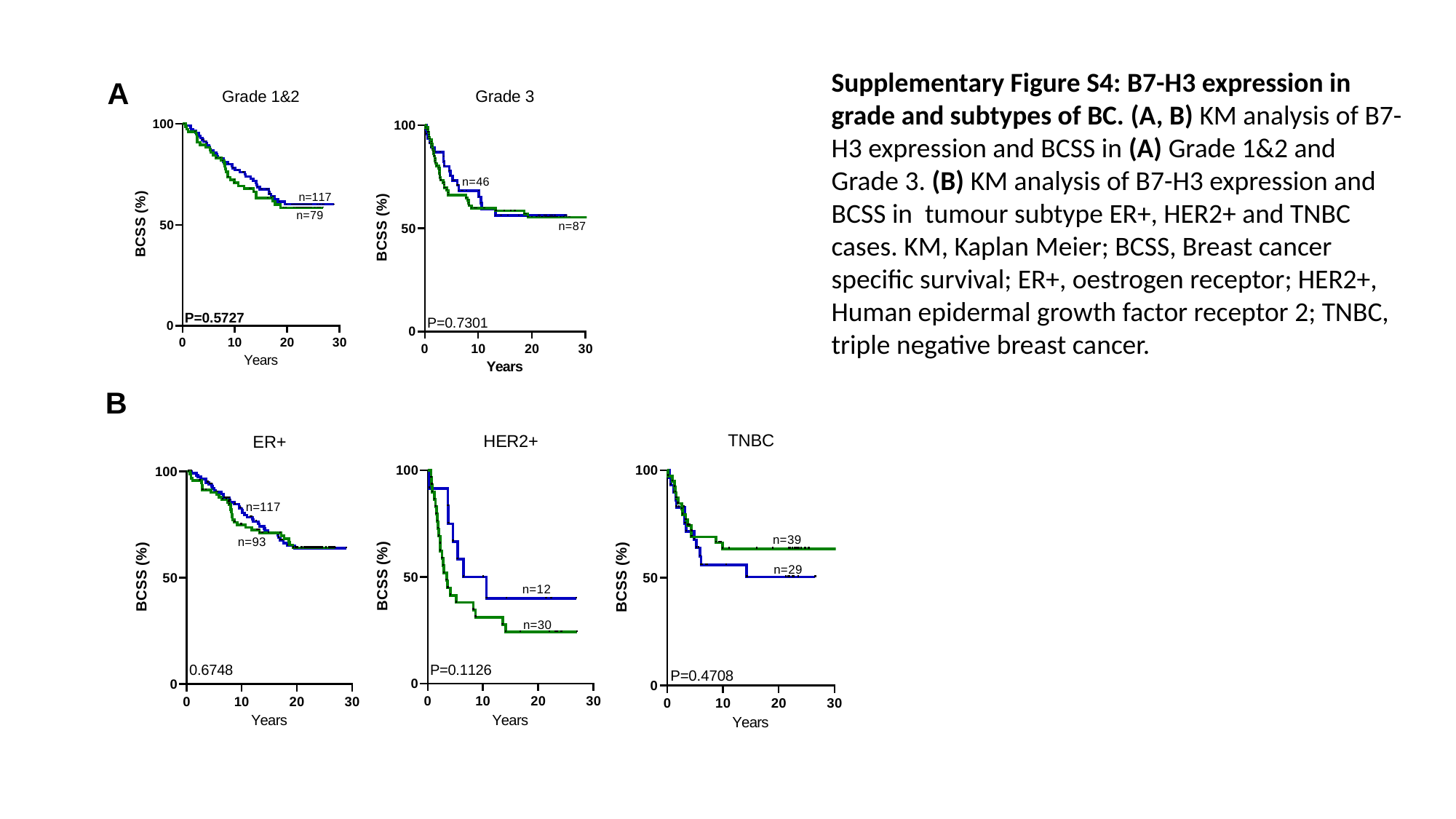

Supplementary Figure S4: B7-H3 expression in grade and subtypes of BC. (A, B) KM analysis of B7-H3 expression and BCSS in (A) Grade 1&2 and Grade 3. (B) KM analysis of B7-H3 expression and BCSS in tumour subtype ER+, HER2+ and TNBC cases. KM, Kaplan Meier; BCSS, Breast cancer specific survival; ER+, oestrogen receptor; HER2+, Human epidermal growth factor receptor 2; TNBC, triple negative breast cancer.
